# Neonatal hypoxia: impacts on the developing mind and brain

**DOI:** 10.3389/fcogn.2025.1565759

**Published:** 2025-02-28

**Authors:** Nafiseh Shabani, Alice Mado Proverbio

**Affiliations:** ^1^Cognitive Electrophysiology Laboratory, Department of Psychology of University of Milano-Bicocca, Milan, Italy; ^2^Neuro-MI Center for Neuroscience, University of Milano-Bicocca, Milan, Italy

**Keywords:** hypoxia, infant, brain, mental process, neural development, neonatal

## Abstract

This review examines the cognitive, emotional, and neurofunctional effects of neonatal hypoxia in both the short and long term. Neonatal hypoxic-ischemic encephalopathy (NHIE) is a critical condition with profound and lasting effects on brain development and function. This mini review examines the structural, cognitive, behavioral, and psychopathological outcomes associated with NHIE, highlighting its impact on neurodevelopment. NHIE is linked to structural abnormalities such as reduced white matter integrity, ventricular enlargement, and damage to key regions including the basal ganglia, hippocampus, and corpus callosum. These changes correlate with long-term impairments in cognition, memory, and motor skills, alongside elevated risks of neurodevelopmental disorders such as autism spectrum disorder (ASD) and attention deficit/hyperactivity disorder (ADHD). Behavioral and emotional challenges, including anxiety, depression, and mood instability, are also prevalent. This review underscores the significant and multifaceted impact of NHIE on neurodevelopmental and behavioral health, emphasizing the importance of developing methodologies to eliminate or minimize neonatal hypoxic states as much as possible.

## Introduction

It has been widely demonstrated in adults that hypoxia significantly impacts neurocognitive function, leading to alterations in behavior and brain activity. Evidence from neuroimaging studies highlights structural and functional changes, shedding light on the mechanisms underlying these deficits (Zani et al., 2024). On the other hand, neonatal hypoxia, a lack of oxygen occurring to the brain before or shortly after birth, is one of the most common complications during childbirth, particularly in cases of complications such as umbilical cord compression or placental abruption. It remains a significant threat to neonatal health, even in developed countries. This condition is particularly critical, as timely and skilled medical intervention within minutes can prevent death or life-long disability.

The severity of hypoxia plays a critical role in neonatal outcomes, as oxygen deprivation can initiate a cascade of cellular dysfunction, including excitotoxicity, oxidative stress, and mitochondrial impairments (Juul and Ferriero, 2014). Neonatal Hypoxic-Ischemic Encephalopathy (NHIE), a severe manifestation of reduced blood and oxygen flow to the brain during pregnancy or birth, often results in significant brain damage (Abusaleem et al., 2024). This condition can lead to acute or subacute brain injury, highlighting its critical impact on neonatal health. The global incidence of NHIE varies within and between countries, in developed countries, the prevalence of HIE is estimated at 1–3 per, 1000 live births (Ristovska et al., 2022). While in developing country, the incidence can be much higher, ranging from 5 to 40 per 1,000 births (Korf et al., 2023). Despite advancements in neonatal care, NHIE remains a major medical concern worldwide. The mortality rate of NHIE is high, as it is one of the leading causes of newborn deaths. In severe cases, around half of affected infants either do not survive or develop severe neurological complications such as cerebral palsy, and cognitive and learning impairment and emotional difficulties (Namusoke et al., 2018; Korf et al., 2023). The consequences of NHIE can be devastating, including lifelong impairments such as autism, cerebral palsy (CP), visual deficits, and other neurological disorders. It is associated with a high risk of brain injury and long-term neurological and neurodevelopmental impairments, including cognitive impairment, behavioral and sensory deficits (Van Handel et al., 2007; Ouwehand et al., 2020; Schreglmann et al., 2020). In addition to mortality, nearly 60% of NHIE cases result in severe disability such as CP (Edoigiawerie et al., 2024). Furthermore, the neonatal brain exhibits regional vulnerability, with the basal ganglia, thalamus, and hippocampus, corpus callosum being particularly prone to damage (Khwaja and Volpe, 2008; Lemmon et al., 2017; Spencer et al., 2023). Long-term neurodevelopmental outcome depends on the severity of NHIE: while severe NHIE significantly increases the risk of mortality and neurodevelopmental issues, mild or moderate NHIE might face a better prognosis and outcomes are more variable. Deficits in the absence of CP or major disability may include intellectual impairments, specific memory and verbal problems, and difficulties in executive functions, behavior, and social competence (Perez et al., 2013). Therapeutic hypothermia (TH) is the standard treatment for moderate to severe NHIE, effectively reducing the risk of death and severe brain damage in infants by 18–24 months and improving cognitive outcomes into school age. Despite variability in outcomes, particularly for moderate NHIE, TH remains a crucial intervention, enhancing survival and mitigating severe disabilities in neonates (Abusaleem et al., 2024; Schreglmann et al., 2020).

NHIE not only affects the infants but also places a heavy burden on their families and society. Global research efforts are crucial to reducing its occurrence and improving treatment outcomes. The present review synthesizes current evidence regarding the effects of NHIE on brain development and cognitive, behavioral and psychopathological outcomes, drawing on recent findings to elucidate underlying mechanisms and therapeutic approaches.

## Methodology

Articles were collected from the following databases: PubMed, SCOPUS, Web of Science, and Google Scholar to identify relevant studies. The search strategy involved Pubmed Medical Subject Heading (MeSH) terms and keywords including “Hypoxic-ischemic encephalopathy,” “Neonatal,” “Cognitive function,” “Hypoxia,” “Brain abnormalities,” “Neurodevelopmental,” “Behavioral ability,” “Mood disorders,” “Autism spectrum disorder (ASD),” and “Attention deficit/hyperactivity disorder (ADHD).” Studies were included based on specific criteria. Eligible studies had to investigate the relationship between neonatal HIE and cognitive impairment, regardless of research design, including participants with a confirmed NHIE diagnosis. Only full-text publications in English that examined human trials were considered. To ensure relevance and up-to-date findings, we mainly focused on recent published papers describing hypoxia-related injuries in different regions of the brain and their consequences and range of severity in NHIE patients. The selected studies were analyzed, and relevant data were extracted to create summary tables providing a qualitative assessment of study components and outcomes. The most suitable approach for synthesizing and interpreting the data for this mini-review was determined after data collection.

### Hypoxia-dependent structural abnormalities in infancy

Table 1 presents the most common neurological damages and structural abnormalities associated with neonatal hypoxia. As noted by Im et al. (2024), cognitive and behavioral challenges are frequently associated with structural anomalies, such as reduced corpus callosum volume, decreased brainstem volume and increased ventricular size, leading to delayed neurodevelopmental outcomes (Im et al., 2024; Huntingford et al., 2024). Atypical signal intensities in the basal ganglia and thalamus strongly predict severe NHIE and adverse neurodevelopmental outcomes at 18–24 months, with deep brain lesions in these regions being key indicators of abnormal neurodevelopment. Furthermore, magnetic resonance imaging (MRI) studies revealed significant changes, including reduced white matter integrity and abnormalities in axonal structures such as the corpus callosum (Bonkowsky and Son, 2018). Perez et al. (2013) found that children with NHIE, even without severe disabilities, faced higher risks of intellectual, verbal, and motor deficits. Their MRI findings revealed that watershed injuries (e.g., damage at the cortical junctions between anterior and middle cerebral artery territories due to reduced blood flow) were strongly associated with poorer verbal and intellectual outcomes, while severe basal ganglia and thalamic injuries correlated with worse prognoses in children with major disabilities. The basal ganglia/thalamus pattern is linked to severe motor and cognitive impairments, whereas the watershed-predominant pattern is a predominant pattern more commonly associated with cognitive deficits in the absence of functional motor impairments (Barnett et al., 2002). Similarly, Dimitrova et al. (2021) reported that increased ventricular volume predicted poorer motor and language outcomes in preterm infants. Erdi-Krausz et al. (2021) further examined school-aged children (ages 5–7) with NHIE, revealing higher rates of minor neurological dysfunction, suboptimal *Movement Assessment Battery for Children-2* (MABC-2) scores (Henderson et al., 1992), and elevated inattention, which inversely correlated with motor performance. Neuromotor challenges, particularly in manual dexterity and balance, were prominent, aligning with parental concerns about motor skills in daily activities.

**Table 1:** Structural abnormalities in NHIE.

**Brain area**	**Damage/abnormality**	**Primary consequence**	**Additional notes**	**References**
Corpus callosum	Reduced volume	Cognitive and behavioral challenges	Highlights structural anomalies as key predictors	Im et al., 2024
Brainstem	Reduced volume	Delayed neurodevelopmental outcomes	Neurodevelopmental delays often tied to motor and cognitive skills	Im et al., 2024; Badawi et al., 2006
Ventricles	Increased size	Poorer motor and language outcomes	Increased ventricular size correlates with neurodevelopmental delays in preterm infants	Im et al. (2024)
Basal Ganglia Thalamus	Atypical signal intensities	Severe motor and cognitive impairments	Strong predictors of adverse outcomes at 18–24 months, especially in NHIE cases	Ouwehand et al., 2020; Perez et al., 2013; Hortigüela et al., 2024
Watershed areas	Watershed injuries	Poor verbal and intellectual outcomes	Typically associated with cognitive impairments in the absence of motor deficits	Perez et al., 2013
Frontal cortex, thalamus, brainstem	Reduced serotonin levels	Increased risk of psychiatric disorders (e.g., schizophrenia, depression)	Neuroinflammation and neuronal degeneration significantly reduce serotonin levels	Wixey et al., 2011; Nosarti et al., 2014
Temporal Lobes (Hippocampus, Amygdala)	Temporal-prefrontal connectivity damage	Disruption in memory, emotional regulation, stress responses	Implicated in psychotic symptoms and early neurodegenerative changes like Alzheimer's	Cannon et al., 2002; Nalivaeva et al., 2018

### Hypoxia-related neurodevelopmental and cognitive disabilities

Edmonds et al. (2020) examined behavioral, cognitive, and neurological outcomes in children without CP at age 2 following NHIE treated with TH, Children with minor neurological signs demonstrated lower cognitive, language, and motor scores and higher scores on *Child Behavior Checklist* (CBCL) (Achenbach and Edelbrock, 1993). They also had increased parent-reported sleep issues and elevated anxiety/depression subscale scores, with trends toward significance in emotional reactivity and somatic complaints. These findings highlight that minor neurological signs are associated with broader developmental like slightly higher autistic trait ratings in *Quantitative Checklist for Autism in Toddlers scores* (Magiati et al., 2015; Edmonds et al., 2020). Furthermore, Edmonds et al. (2021) later found how memory impairments are also a prominent feature, with significant difficulties observed both in objective measures, such as the *Rivermead Behavioral Memory Test* (Wilson et al., 1991), and in real-life scenarios, where parents and teachers report frequent issues, such as forgetting instructions and losing track of tasks (Edmonds et al., 2021). In a cohort study conducted by Hortigüela et al. (2024), neurodevelopmental outcomes were evaluated at 3 years of age in children that suffered from moderate to severe NHIE treated with TH. Their findings indicated that a considerable proportion of survivors experienced adverse outcomes, including sensory impairments, epilepsy, cerebral palsy, and developmental delays. They identified that 14.2% of patients exhibited minor motor impairments (MMI), reflecting a shift from severe to milder motor impairments due to the effectiveness of TH. Notably, half of the patients with MMI also experienced broader neurodevelopmental delays, such as motor and language challenges (Martinez et al., 2014). While less severe than cerebral palsy, MMI affected daily activities, manifesting as subtle coordination difficulties and delayed motor milestones. Schreglmann et al. (2020) reviewed of follow-up focused on cohorts aged 4 years and older found that many children with NHIE who do not develop cerebral palsy (CP) are at a heightened risk of cognitive impairments. These challenges are most pronounced in domains such as attention, language, and executive functions, with minimal evidence available concerning associated behavioral issues.

Later in a longitudinal study indicated that children with an average age of 6.25 years (range 5.5, 7.33) who experienced NHIE are at increased risk for cognitive impairments, including deficits in working memory, processing speed, problem-solving, and attentional problems, with intelligence emerging as a distinguishing factor between NHIE survivors and their peers (Cainelli et al., 2021). Then Erdi-Krausz et al. (2021) further examined school-aged children (ages 5–7) with NHIE, revealing higher rates of minor neurological dysfunction, suboptimal *Movement Assessment Battery for Children-2* (MABC-2) scores, and elevated inattention, which inversely correlated with motor performance. Neuromotor challenges, particularly in manual dexterity and balance, were prominent, aligning with parental concerns about motor skills in daily activities.

In another study, children aged 6–8 years who underwent TH for NHIE without developing CP were the focus of the study by Lee-Kelland et al. (2020). They revealed cognitive deficits in this group, with IQ scores averaging 14 points lower, particularly in perceptual reasoning and processing speed, while verbal IQ was less affected, thus emphasizing the need for peer-based comparisons rather than relying solely on standardized test norms, given the higher proportion of subnormal IQ scores. Based on *Movement Assessment Battery for Children-2* (MABC-2) scores, Motor impairments were also notable, NHIE had significantly lower total MABC-2 scores than controls, indicating overall poorer motor performance and delayed independent walking.

Perez et al. (2013) examined a cohort of school and teenage age children ranging from 8 to 15 years old (on average 11 years) with a history of NHIE (not treated with TH) who had survived without developing CP. Despite the absence of major disabilities such as severe CP or intellectual disability, children with NHIE were at increased risk for intellectual deficits, with lower IQ scores, particularly affecting verbal and performance IQ, and higher rates of learning disabilities. Motor impairments were also more prevalent, with up to a threefold increase in poor performance (Perez et al., 2013).

### Psychiatric and psychopathological deficits following NHIE

Among the most common psychiatric deficits (listed in Table 2) are sleep and mood disorders, personality disorders, ADHD, aggressiveness, anxiety, depression, autism and schizophrenia. For example, Edmonds et al. (2020) examined behavioral, cognitive, and neurological outcomes in a clinical cohort of children without CP at age 2 years following NHIE. Their findings suggest that while performance on the standardized assessment comprising developmental measures was comparable to the general population, children who suffered from neonatal hypoxia exhibited higher rates of externalizing problems and sleep difficulties (Edmonds et al., 2020). Ding et al. (2016) investigated the sleep qualities of 2-year-old NHIE children with varying degrees of NHIE conditions and identified distinct sleep-related issues associated with mild and moderate hypoxia and found that children with moderate NHIE were more prone to sleep initiation and maintenance difficulties, as well as sleep-related breathing problems. Again, children with mild hypoxia experienced challenges maintaining regular circadian rhythms (Ding et al., 2016).

**Table 2:** Common NHIE-related psychiatric deficits.

**Severity of NHIE**	**Psychiatric disorder**	**Reasons**	**Reference**
Moderate/severe	Anxiety and depression and aggressive behavior	Promotes inflammation, mitochondrial dysfunction, and serotonin deficiency	Hortigüela et al., 2024; Burtscher et al., 2022
Moderate/severe	Higher rates of externalizing problems and sleep difficulties	–	Edmonds et al., 2020
Moderate/severe	Increased risk of mood disorders, particularly anxiety and depression	–	Álvarez-García et al., 2022
Severe	Attention-deficit/hyperactivity disorder (ADHD)	–	Getahun et al., 2013
Moderate/severe	Autism spectrum disorder (ASD)	Reduces serotonin (5-HT) levels in frontal cortex, thalamus, and brainstem. Promotes neuroinflammation and serotonergic neural degeneration	Getahun et al., 2017; Wixey et al., 2011
Moderate	Autism spectrum disorder (ASD)	Reduced white matter integrity and abnormalities in axonal structures such as the corpus callosum	Bonkowsky and Son, 2018
Severe	Depression and schizophrenia	Reduced gray matter. Increased cerebrospinal fluid in the temporal lobes. Ventricular enlargement	Nosarti et al., 2014
Moderate/mild	Disrupts memory, emotional regulation, and stress responses	Damage to the hippocampus and amygdala	Cannon et al., 2002
Moderate/severe	Hyperactivity, anxiety, and depression	–	Lee-Kelland et al., 2020
Moderate/mild	sleep-related issues	–	Ding et al., 2016

The study by Hortigüela et al. (2024) reported a higher prevalence of behavioral and emotional challenges in 3 years of age in children compared to their peers. Emotional difficulties, such as anxiety and depression, were markedly more frequent among children with NHIE. This aligns with earlier epidemiological studies linking this to increased anxiety, depressive symptoms, and mood instability. The interplay between hypoxia and mental stress further contributes to anxiety and depressive disorders by disrupting the hypoxia inducible factor pathway, promoting inflammation, mitochondrial dysfunction, and serotonin deficiency (Burtscher et al., 2022; Lestón Pinilla et al., 2021). Consistent with these findings, Álvarez-García et al. (2022) underscored the socio-emotional vulnerabilities observed in children aged 3–6 years who survived NHIE treated with TH. Their study revealed an elevated risk of mood disorders, particularly anxiety and depression, as assessed through the *Child Behavior Checklist* (CBCL) and the *Preschool Symptom Self-Report* (Martini et al., 1990). Survivors of NHIE exhibited a higher prevalence of anxious/depressive symptoms and aggressive behaviors, with NHIE-affected children demonstrating significantly higher scores for these traits on the CBCL. The increased incidence of mood disorders in NHIE survivors aligns with patterns observed in adults who experience acute ischemic brain injury, where the prevalence of mood disorders, particularly depression and anxiety, is notably heightened (Álvarez-García et al., 2022). To investigate the association between perinatal factors and autism spectrum disorders (ASD), a retrospective cohort study by Getahun et al. (2017) examined children aged 3–17 years. The findings revealed that children with ASD were significantly more likely to have experienced perinatal complications compared to their neurotypical peers. In an earlier study by Getahun et al. (2013), which focused on children aged 5–11 years, a correlation was identified between NHIE conditions and an elevated risk of attention deficit/hyperactivity disorder (ADHD), another neurodevelopmental disorder. This study highlighted that NHIE, particularly birth asphyxia and respiratory distress syndrome, is independently associated with ADHD, with the association being strongest in preterm births. Although ASD and ADHD exhibit distinct characteristics, they share overlapping etiological factors, neurological features, symptoms, and comorbidities (Getahun et al., 2013, 2017).

This aligns with findings reported by Lee-Kelland et al. (2020), who investigated children aged 6–8 years treated with TH for NHIE and who did not develop CP behavioral and emotional difficulties emerged as significant concerns, with these children exhibiting heightened emotional challenges and behavioral issues compared to their peers. Parents reported elevated rates of hyperactivity, anxiety, and depression, consistent with prior studies on non-cooled children with moderate NHIE.

Dysregulated serotonergic systems, often implicated in behavioral disorders such as depression, anxiety, and developmental conditions like ASD and sudden infant death syndrome, may play a significant role. NHIE has been shown to markedly reduce serotonin (5-HT) levels in key brain regions, including the frontal cortex, thalamus, and brainstem, primarily due to neuroinflammation and serotonergic neuronal degeneration (Wixey et al., 2011). Bonkowsky and Son (2018), in their review, highlighted the profound impact of NHIE on neural connectivity, emphasizing its role in behavioral changes and the heightened risk of neurodevelopmental disorders, such as ASD. They presented evidence of altered electroencephalogram patterns in individuals living at high altitudes (see also Zani et al., 2024) and disruptions in neural connectivity associated with NHIE.

Overall, NHIE is associated with a heightened risk of psychiatric disorders, including depression and schizophrenia, later in life (Hortigüela et al., 2024; Nosarti et al., 2014). Cannon et al. (2002) strongly linked NHIE to structural brain changes, such as reduced gray matter, increased cerebrospinal fluid in the temporal lobes, and ventricular enlargement—a hallmark of schizophrenia. He further reported that damage to the temporal lobes (housing critical structures such as the hippocampus and amygdala), disrupts memory, emotional regulation, and stress responses, contributing to psychotic symptoms. NHIE induced impairment of temporal-prefrontal connectivity exacerbates executive dysfunction and auditory hallucinations (Cannon et al., 2002). Finally, Nalivaeva et al. (2018) emphasize that prenatal hypoxia during critical brain development stages disrupts cognitive function, reduces plasticity, and increases the risk of neurodegenerative disorders by impairing neuronal connections, particularly in the cortex and hippocampus. These effects are compounded by dendritic spine reductions, which impair synaptic connections and are linked to early neurodegenerative changes, including those associated with Alzheimer's disease (Nalivaeva et al., 2018).

## Conclusions

Neonatal hypoxic-ischemic encephalopathy (NHIE) profoundly impacts neurodevelopment, causing extensive structural and functional disruptions in the brain. Key structural abnormalities, such as damage to the basal ganglia, hippocampus, and corpus callosum, contribute to the cognitive and motor deficits commonly observed in affected individuals. Children with NHIE are at significantly increased risk for neurodevelopmental disorders, including autism spectrum disorder and attention deficit/hyperactivity disorder, alongside intellectual impairments, memory deficits, and delayed motor development. Moreover, behavioral and emotional challenges, such as anxiety, depression, and mood instability, further compound the long-term prognosis. These findings emphasize the pivotal role of NHIE in shaping developmental trajectories and highlight the need for a deeper understanding of its multifaceted impacts to better support affected individuals.

## References

[B1] Abusaleem M. Y. Ebrahim M. E. E. Hamed N. F. Eladwy M. F. M. (2024). A systematic review of the relationship between neonatal hypoxic-ischemic encephalopathy and long-term cognitive outcomes: where do we stand? Cureus 16:e68227. 10.7759/cureus.68227 39347282 PMC11439448

[B2] Achenbach T. M. Edelbrock C. S. (1993). Manual for the Child Behavior Checklist and Revised Child Behavior Profile. Burlington, VT: University of Vermont.

[B3] Álvarez-García M. Cuellar-Flores I. Sierra-García P. Martínez-Orgado J. (2022). Mood disorders in children following neonatal hypoxic-ischemic encephalopathy. PLoS ONE 17:e0263055. 10.1371/journal.pone.0263055 35089978 PMC8797213

[B4] Badawi N. Dixon G. Felix J. F. Keogh J. M. Petterson B. Stanley F. J.. (2006). Autism following a history of newborn encephalopathy: more than a coincidence? Dev. *Med. Child Neurol*. 48, 85–89. 16417661 10.1017/S001216220600020X

[B5] Barnett A. Mercuri E. Rutherford M. Haataja L. Frisone M. F. Henderson S.. (2002). Neurological and perceptual-motor outcome at 5-6 years of age in children with neonatal encephalopathy: relationship with neonatal brain MRI. Neuropediatrics 33, 242–248. 10.1055/s-2002-36737 12536366

[B6] Bonkowsky J. L. Son J. H. (2018). Hypoxia and connectivity in the developing vertebrate nervous system. Dis. Models Mech. 11:037127. 10.1242/dmm.037127 30541748 PMC6307895

[B7] Burtscher J. Niedermeier M. Hüfner K. van den Burg E. Kopp M. Stoop R.. (2022). The interplay of hypoxic and mental stress: implications for anxiety and depressive disorders. Neurosci. Biobehav. Rev. 138:104718. 10.1016/j.neubiorev.2022.104718 35661753

[B8] Cainelli E. Vedovelli L. Mastretta E. Gregori D. Suppiej A. Bisiacchi P. S.. (2021). Long-term outcomes after neonatal hypoxic-ischemic encephalopathy in the era of therapeutic hypothermia: a longitudinal, prospective, multicenter case-control study in children without overt brain damage. Children 8:1076. 10.3390/children8111076 34828791 PMC8625352

[B9] Cannon T. D. van Erp T. G. Rosso I. M. Huttunen M. Lönnqvist J. Pirkola T.. (2002). Fetal hypoxia and structural brain abnormalities in schizophrenic patients, their siblings, and controls. *Arch. Gen. Psychiatry* 59, 35–41. 11779280 10.1001/archpsyc.59.1.35

[B10] Dimitrova R. Arulkumaran S. Carney O. Chew A. Falconer S. Ciarrusta J.. (2021). Phenotyping the preterm brain: characterizing individual deviations from normative volumetric development in two large infant cohorts. *Cereb. Cortex*. 31, 3665–3677. 33822913 10.1093/cercor/bhab039 PMC8258435

[B11] Ding X. Cheng Z. Sun B. Huang J. Wang L. Han X.. (2016). Distinctive sleep problems in children with perinatal moderate or mild hypoxic-ischemia. Neurosci. Lett. 614, 60–64. 10.1016/j.neulet.2015.12.061 26762786

[B12] Edmonds C. J. Cianfaglione R. Cornforth C. Vollmer B. (2021). Children with neonatal Hypoxic Ischaemic Encephalopathy (HIE) treated with therapeutic hypothermia are not as school ready as their peers. Acta Paediatr. 110, 2756–2765. 10.1111/apa.16002 34160861

[B13] Edmonds C. J. Helps S. K. Hart D. Zatorska A. Gupta N. Cianfaglione R.. (2020). Minor neurological signs and behavioral function at age 2 years in neonatal hypoxic ischemic encephalopathy (HIE). Eur. J. Pediatr. Neurol. 27, 78–85. 10.1016/j.ejpn.2020.04.003 32327390

[B14] Edoigiawerie S. Henry J. Issa N. David H. (2024). A systematic review of EEG and MRI features for predicting long-term neurological outcomes in cooled neonates with Hypoxic-Ischemic Encephalopathy (HIE). Cureus 16:e71431. 10.7759/cureus.71431 39539899 PMC11558949

[B15] Erdi-Krausz G. Rocha R. Brown A. Myneni A. Lennartsson F. Romsauerova A.. (2021). Neonatal hypoxic-ischaemic encephalopathy: motor impairment beyond cerebral palsy. *Eur. J. Paediatr. Neurol*. 35, 74–81. 34666231 10.1016/j.ejpn.2021.10.005

[B16] Getahun D. Fassett M. J. Peltier M. R. Wing D. A. Xiang A. H. Chiu V.. (2017). Association of perinatal risk factors with autism spectrum disorder. Am. J. Perinatol. 7, 295–304. 10.1055/s-0036-1597624 28099978

[B17] Getahun D. Rhoads G. G. Demissie K. Lu S. E. Quinn V. P. Fassett M. J.. (2013). In utero exposure to ischemic-hypoxic conditions and attention-deficit/hyperactivity disorder. Pediatrics 131, e53–e61. 10.1542/peds.2012-1298 23230063

[B18] Henderson S. E. Sugden D. Barnett A. L. (1992). Movement Assessment Battery for Children-2. Research in Developmental Disabilities. London: The Psychological Corporation.

[B19] Hortigüela M. M. Martínez-Biarge M. Conejo D. Vega-del-Val C. Arnaez J. Arahip G. (2024). Motor, cognitive and behavioural outcomes after neonatal hypoxic-ischaemic encephalopathy. An. Pediatr. 100, 104–114. 10.1016/j.anpede.2024.01.009 38331678

[B20] Huntingford S. L. Boyd S. M. McIntyre S. J. Goldsmith S. C. Hunt R. W. Badawi N.. (2024). Long-term outcomes following hypoxic ischemic encephalopathy. Clin. Perinatol. 51, 683–709. 10.1016/j.clp.2024.04.008 39095104

[B21] Im S. A. Tomita E. Oh M. Y. Kim S. Y. Kang H. M. Youn Y. A.. (2024). Volumetric changes in brain MRI of infants with hypoxic-ischemic encephalopathy and abnormal neurodevelopment who underwent therapeutic hypothermia. Brain Res. 1825:148703. 10.1016/j.brainres.2023.148703 38101694

[B22] Juul S. E. Ferriero D. M. (2014). Pharmacologic neuroprotective strategies in neonatal brain injury. Clin. Perinatol. 41, 119–131. 10.1016/j.clp.2013.09.004 24524450 PMC3929237

[B23] Khwaja O. Volpe J. J. (2008). Pathogenesis of cerebral white matter injury of prematurity. Arch. Dis. Child. Fetal. Neonatal. Ed. 93, F153–F161. 10.1136/adc.2006.108837 18296574 PMC2569152

[B24] Korf J. M. McCullough L. D. Caretti V. (2023). A narrative review on treatment strategies for neonatal hypoxic ischemic encephalopathy. Transl. Pediatr. 12:1552. 10.21037/tp-23-253 37692539 PMC10485647

[B25] Lee-Kelland R. Jary S. Tonks J. Cowan F. M. Thoresen M. Chakkarapani E.. (2020). School-age outcomes of children without cerebral palsy cooled for neonatal hypoxic–ischaemic encephalopathy in 2008–2010. Arch. Dis. Child. Fetal. Neonatal. Ed. 105, 8–13. 10.1136/archdischild-2018-316509 31036702

[B26] Lemmon M. E. Wagner M. W. Bosemani T. Carson K. A. Northington F. J. Huisman T. A.. (2017). Diffusion tensor imaging detects occult cerebellar injury in severe neonatal hypoxic-ischemic encephalopathy. Dev. Neurosci. 39, 207–214. 10.1159/000454856 28095379 PMC5607011

[B27] Lestón Pinilla L. Ugun-Klusek A. Rutella S. De Girolamo L. A. (2021). Hypoxia signaling in parkinson's disease: there is use in asking what HIF? Biology 10:723. 10.3390/biology10080723 34439955 PMC8389254

[B28] Magiati I. Goh D. A. Lim S. J. Gan D. Z. Q. Leong J. C. L. Allison C.. (2015). The psychometric properties of the Quantitative-Checklist for Autism in Toddlers (Q-CHAT) as a measure of autistic traits in a community sample of Singaporean infants and toddlers. Mol. Autism 6, 1–14. 10.1186/s13229-015-0032-1 26124950 PMC4484636

[B29] Martinez C. Carneiro L. Vernier L. Cesa C. Guardiola A. Vidor D.. (2014). Language in children with neonatal hypoxic-ischemic encephalopathy. Int. Arch. Otorhinolaryngol. 18, 255–259. 10.1055/s-0034-1366976 25992102 PMC4297029

[B30] Martini D. R. Strayhorn J. M. Puig-Antich J. (1990). A symptom self-report measure for preschool children. J. Am. Acad. Child. Adolesc. Psychiatry 29, 594–600. 10.1097/00004583-199007000-00013 2387794

[B31] Nalivaeva N. N. Turner A. J. Zhuravin I. A. (2018). Role of prenatal hypoxia in brain development, cognitive functions, and neurodegeneration. Front. Neurosci. 12:825. 10.3389/fnins.2018.00825 30510498 PMC6254649

[B32] Namusoke H. Nannyonga M. M. Ssebunya R. Nakibuuka V. K. Mworozi E. (2018). Incidence and short-term outcomes of neonates with hypoxic ischemic encephalopathy in a Peri Urban teaching hospital, Uganda: a prospective cohort study. Matern. Health Neonatol. Perinatol. 4, 1–6. 10.1186/s40748-018-0074-4 29556412 PMC5840790

[B33] Nosarti C. Nam K. W. Walshe M. Murray R. M. Cuddy M. Rifkin L.. (2014). Preterm birth and structural brain alterations in early adulthood. Neuroimage Clin. 6, 180–191. 10.1016/j.nicl.2014.08.005 25379430 PMC4215396

[B34] Ouwehand S. Smidt L. C. Dudink J. Benders M. J. de Vries L. S. Groenendaal E.. (2020). Predictors of outcomes in hypoxic-ischemic encephalopathy following hypothermia: a meta-analysis. Neonatology 117, 411–427. 10.1159/000505519 32235122

[B35] Perez A. Ritter S. Brotschi B. Werner H. Caflisch J. Martin E.. (2013). Long-term neurodevelopmental outcome with hypoxic-ischemic encephalopathy. J. Pediatr. 163, 454–459. 10.1016/j.jpeds.2013.02.003 23498155

[B36] Ristovska S. Stomnaroska O. Danilovski D. (2022). Hypoxic ischemic encephalopathy (HIE) in term and preterm infants. Prilozi 43, 77–84. 10.2478/prilozi-2022-0013 35451288

[B37] Schreglmann M. Ground A. Vollmer B. Johnson M. J. (2020). Systematic review: long-term cognitive and behavioral outcomes of neonatal hypoxic–ischaemic encephalopathy in children without cerebral palsy. Acta Paediatr. 109, 20–30. 10.1111/apa.14821 31002422

[B38] Spencer A. P. Lee-Kelland R. Brooks J. C. Jary S. Tonks J. Cowan F. M.. (2023). Brain volumes and functional outcomes in children without cerebral palsy after therapeutic hypothermia for neonatal hypoxic-ischaemic encephalopathy. Dev. Med. Child Neurol. 65, 367–375. 10.1111/dmcn.15369 35907252 PMC10087533

[B39] Van Handel M. Swaab H. De Vries L. S. Jongmans M. J. (2007). Long-term cognitive and behavioral consequences of neonatal encephalopathy following perinatal asphyxia: a review. Eur. J. Pediatr. 166, 645–654. 10.1007/s00431-007-0437-8 17426984 PMC1914268

[B40] Wilson B. A. Ivani-Chalian R. Aldrich F. (1991). The Rivermead behavioural memory test for children aged 5 to 10 years. Bury St. Edmunds: Thames Valley Test Company.

[B41] Wixey J. A. Reinebrant H. E. Buller K. M. (2011). Inhibition of neuroinflammation prevents injury to the serotonergic network after hypoxia-ischemia in the immature rat brain. J. Neuropathol. Exp. Neurol. 70, 23–35. 10.1097/NEN.0b013e3182020b7b 21157380

[B42] Zani A. Dishi Y. Proverbio A. M. (2024). From oxygen shortage to neurocognitive challenges: behavioral patterns and imaging insights. Front. Cogn. 3:1468306. 10.3389/fcogn.2024.1468306 PMC13281051 42339455

